# Small Intestinal Bacterial Overgrowth Following Gastrointestinal Cancer Surgery: Current Evidence and Future Perspectives

**DOI:** 10.1002/ags3.70291

**Published:** 2026-10-01

**Authors:** Toru Aoyama, Haruhiko Cho, Takaki Yoshikawa

**Affiliations:** ^1^ Department of Gastrointestinal Surgery Yokohama City University Yokohama Japan; ^2^ Department of Gastric Surgery Tokyo Metropolitan Cancer and Infectious Diseases Center Komagome Hospital Tokyo Japan

**Keywords:** colorectal surgery, esophagectomy, gastrectomy, gastrointestinal cancer, small intestinal bacterial overgrowth

## Abstract

Small intestinal bacterial overgrowth (SIBO) has recently gained increasing attention as a potential cause of postoperative gastrointestinal symptoms following gastrointestinal cancer surgery. Surgical procedures can disrupt normal gastrointestinal physiology through anatomical reconstruction, impaired motility, reduced gastric acid secretion, vagal nerve injury, and alterations in the gut microbiota, thereby predisposing patients to bacterial overgrowth. Although positive breath tests for SIBO have frequently been reported after esophageal, gastric, and colorectal cancer surgery, the reported positivity rates vary widely because of differences in patient selection, surgical procedures, and diagnostic methods. Importantly, several studies evaluated selected symptomatic or referred patients; therefore, these rates should not necessarily be interpreted as the true prevalence of SIBO in the overall postoperative population. Breath testing is widely used because of its simplicity and non‐invasive nature; however, altered gastrointestinal anatomy and accelerated postoperative transit may affect test specificity and potentially produce false‐positive results. Evidence regarding treatment specifically in patients after gastrointestinal cancer surgery remains limited, and most data regarding rifaximin and other therapeutic approaches are extrapolated from general SIBO populations. This review summarizes the current evidence regarding the epidemiology, pathophysiology, diagnosis, and treatment of SIBO following gastrointestinal cancer surgery. We discuss future perspectives, including standardization of diagnostic criteria, optimization of breath testing, microbiome‐based approaches, refinement of rifaximin therapy, and strategies for recurrence prevention. A better understanding of SIBO may improve the recognition and management of postoperative gastrointestinal symptoms and nutritional abnormalities; however, whether SIBO‐directed management improves body composition, treatment tolerance, postoperative recovery, or long‐term outcomes remains to be established.

## Abstract 2

Small intestinal bacterial overgrowth (SIBO) may contribute to gastrointestinal symptoms after gastrointestinal cancer surgery, but reported breath‐test positivity rates are highly variable and may overestimate true prevalence because of patient selection, altered anatomy, and accelerated postoperative transit. Current evidence is limited and heterogeneous, particularly for pancreatobiliary surgery, and most treatment data are extrapolated from general SIBO populations rather than postoperative cancer patients. Standardized diagnostic approaches and prospective studies are needed to clarify the clinical significance of SIBO and establish evidence‐based management strategies in this setting.

## 1 Introduction

According to GLOBOCAN 2022, an estimated 20.0 million new cancer cases and 9.7 million cancer‐related deaths occurred worldwide [1]. Curative surgery remains the cornerstone of treatment for patients with gastrointestinal malignancies. Although surgical treatment plays a central role in achieving cure, various postoperative gastrointestinal symptoms, including abdominal pain, abdominal bloating, diarrhea, nausea, and vomiting, frequently develop after surgery [2, 3]. These symptoms may result from multiple factors, such as reduced gastric acid secretion, impaired small intestinal motility, alterations in the gut microbiota, surgically induced changes in gastrointestinal anatomy, and reduced bile secretion capacity [4, 5]. However, the underlying mechanisms of these symptoms are often poorly understood and established diagnostic and therapeutic strategies remain limited.

Recently, small intestinal bacterial overgrowth (SIBO) has attracted increasing attention as a potential cause of postoperative gastrointestinal symptoms in patients undergoing gastrointestinal surgery [6, 7]. SIBO is characterized by excessive bacterial proliferation within the small intestine and may contribute to a wide range of gastrointestinal and nutritional disorders. However, evidence regarding the frequency of positive SIBO testing, diagnostic accuracy, clinical significance, and management of SIBO after gastrointestinal cancer surgery remains limited. A recent review by Mechlińska et al. comprehensively addressed SIBO and intestinal methanogen overgrowth in patients with gastrointestinal malignancies [8]. The present review differs in its specific focus on patients after gastrointestinal cancer surgery. In particular, we emphasize surgery‐induced anatomical and physiological alterations, surgery‐specific limitations in the interpretation of breath testing, and practical considerations for the evaluation and management of postoperative gastrointestinal symptoms.

Although the number of postoperative studies remains limited, several reports published in recent years have added clinically relevant evidence. Since 2022, studies have evaluated SIBO or breath‐test positivity after oesophagogastric cancer resection, gastrectomy, right‐sided hemicolectomy for colorectal cancer, and pancreatic resection [9, 10, 11, 12]. These studies have also highlighted important unresolved issues, including the effects of patient selection, altered gastrointestinal transit, overlapping postoperative disorders, and the limited direct evidence for treatment. Accordingly, an updated surgery‐focused synthesis is warranted not because the evidence base is already extensive, but because recently available data allow a more critical appraisal of the clinical significance and limitations of SIBO testing after gastrointestinal cancer surgery. Thus, this review is intended to provide a clinically oriented framework particularly relevant to surgeons and multidisciplinary teams involved in postoperative survivorship care.

To optimize the diagnosis and treatment of SIBO after gastrointestinal cancer surgery, it is important to understand the current evidence regarding its epidemiology, pathophysiology, diagnostic approaches, and therapeutic strategies. Therefore, this review summarizes the current evidence on SIBO following esophageal, gastric, colorectal, pancreatic, and biliary cancer surgery, with a particular focus on its definition, incidence, diagnosis, treatment, and future perspectives. Because direct evidence regarding SIBO after pancreatobiliary cancer surgery remains limited, the strength and clinical implications of the available evidence are considered separately.

### 1.1 Literature Search and Study Selection

A literature search was conducted using PubMed/MEDLINE from database inception through July 1, 2026, and the final search was performed on July 1, 2026. No language restrictions were applied. The complete PubMed/MEDLINE search strategy is provided in Table S1. The reference lists of eligible articles and relevant reviews were also examined to identify additional potentially eligible studies. Directly relevant non‐PubMed‐indexed reports identified through reference‐list screening and manual citation tracking were also considered eligible if they met the same predefined eligibility criteria. The search terms included combinations of “small intestinal bacterial overgrowth,” “SIBO,” “esophagectomy,” “gastrectomy,” “gastric cancer,” “colorectal surgery,” “colorectal cancer,” “pancreatic cancer,” “pancreatectomy,” “pancreaticoduodenectomy,” “biliary cancer,” “cholangiocarcinoma,” “biliary surgery,” and “gastrointestinal cancer.” Primary studies were considered eligible for the postoperative cancer‐specific evidence synthesis if they included patients who had undergone surgery for a gastrointestinal malignancy and evaluated the occurrence, diagnosis, clinical significance, or treatment of SIBO using breath testing, small intestinal aspirate culture, or other predefined diagnostic methods. Case series were considered eligible for qualitative synthesis but were not used to estimate population‐level prevalence. Studies involving exclusively non‐surgical populations, surgery for benign disease, or cancer patients without extractable postoperative surgical data were excluded from the cancer‐specific evidence synthesis. Relevant clinical guidelines, consensus statements, systematic reviews, and meta‐analyses were additionally reviewed to provide background information on the definition, diagnosis, and treatment of SIBO. Titles and abstracts identified by the search were screened by the first author, followed by full‐text assessment of potentially eligible studies. Uncertainties regarding eligibility were resolved through discussion among the authors. Reports from the same institution or research group were examined for potentially overlapping study populations, and overlapping or non‐cancer‐specific cohorts were not treated as independent cancer‐specific evidence. Because of the limited number of eligible studies and substantial heterogeneity in patient selection, surgical procedures, diagnostic methods, and outcome definitions, a narrative review approach was adopted rather than a quantitative meta‐analysis.

### 1.2 Methodological Quality Assessment

The methodological quality of studies specifically evaluating SIBO after gastrointestinal cancer surgery was assessed using the Joanna Briggs Institute critical appraisal tools appropriate to each study design. The assessment focused on patient selection and representativeness, definition and ascertainment of SIBO, completeness of outcome assessment, identification and management of potential confounding factors, and appropriateness of statistical analyses. Particular attention was paid to whether the study population represented an unselected postoperative cohort or consisted of patients selected because of gastrointestinal symptoms or referral for SIBO testing. The latter design was considered to have an increased risk of selection bias when breath‐test positivity rates were interpreted as estimates of the prevalence of SIBO in the overall postoperative population. In addition, variation in breath‐test substrates, diagnostic thresholds, and postoperative gastrointestinal anatomy was considered an important source of methodological heterogeneity. Detailed methodological characteristics of the included postoperative studies, including sampling frame, indication for testing, postoperative interval, breath‐test protocol, PPI exposure, and assessment of relevant confounders, are summarized in Table S2.

## 2 Pathophysiology of SIBO

The pathophysiology of SIBO is complex and multifactorial. Several mechanisms, including impaired gastrointestinal motility, reduced gastric acid secretion, anatomical alterations, and disruption of the gut microbiota, have been proposed to contribute to the development of SIBO [6, 13]. Among patients who undergo gastrointestinal surgery, postoperative anatomical changes and motility disorders are considered major contributors to bacterial overgrowth. Impaired gastrointestinal motility frequently occurs after gastrointestinal cancer surgery because of surgical manipulation, vagal nerve injury, and reconstruction of the gastrointestinal tract. Postoperative dysmotility may lead to intestinal stasis, thereby promoting bacterial overgrowth within the small intestine [14]. In esophageal cancer surgery, reconstruction is typically performed using a gastric conduit, which may alter normal gastrointestinal motility. In gastric cancer surgery, the most common reconstruction methods include Billroth I, Billroth II, and Roux‐en‐Y reconstruction. Among these procedures, Billroth II and Roux‐en‐Y reconstruction may increase the risk of SIBO because of intestinal stasis and blind loop formation. In colorectal cancer surgery, intestinal continuity is generally restored by primary anastomosis, which preserves normal bowel continuity and avoids blind loop formation. However, right‐sided colectomy with resection of the ileocecal valve may predispose patients to SIBO [15]. The ileocecal valve functions as a mechanical barrier preventing retrograde migration of colonic bacteria into the small intestine. Therefore, loss of this barrier may facilitate bacterial colonization of the small intestine and contribute to the development of SIBO. Reduced gastric acid secretion is another important mechanism contributing to the development of SIBO. Gastric acid serves as a physiological barrier against ingested microorganisms and prevents excessive bacterial colonization of the upper gastrointestinal tract. Therefore, suppression of gastric acid secretion may facilitate bacterial proliferation within the small intestine. Patients undergoing gastrectomy are particularly susceptible to SIBO because gastric acid secretion is markedly reduced after surgery. In addition, vagotomy, which is frequently performed during gastric cancer surgery, may further impair gastric secretion and gastrointestinal motility, thereby creating a favorable environment for bacterial overgrowth. Furthermore, proton pump inhibitors (PPIs), which are commonly prescribed after gastrointestinal surgery, have been associated with an increased risk of SIBO [16]. Several studies have suggested that long‐term acid suppression may alter the gastrointestinal microbial environment and promote bacterial colonization of the small intestine. Gut microbiota dysbiosis is another important mechanism involved in the development of SIBO [4, 17]. The gastrointestinal tract harbors a complex microbial ecosystem that contributes to host metabolism, immune regulation, and maintenance of intestinal homeostasis. Disruption of this microbial balance may promote excessive bacterial colonization of the small intestine and contribute to the development of SIBO. Gastrointestinal surgery can induce substantial alterations in the gut microbiota through anatomical reconstruction, perioperative antibiotic exposure, changes in nutrient flow, and alterations in bile acid metabolism. Previous studies have demonstrated that gastrectomy and esophagectomy are associated with significant shifts in microbial composition, including increases in facultative anaerobic bacteria and reductions in microbial diversity. Such postoperative dysbiosis may impair colonization resistance against pathogenic microorganisms and facilitate bacterial overgrowth within the small intestine. Furthermore, SIBO itself may exacerbate dysbiosis through abnormal fermentation, nutrient malabsorption, and mucosal inflammation, creating a vicious cycle that perpetuates gastrointestinal symptoms. In addition to these mechanisms, impaired mucosal immunity and nutritional deficiencies may also contribute to the development of SIBO. Surgical stress, malnutrition, and cancer‐related immune dysfunction can weaken intestinal barrier function and host defense mechanisms, thereby facilitating bacterial proliferation within the small intestine. The major procedure‐specific anatomical and physiological changes and their putative mechanisms contributing to SIBO are summarized in Table 1.

**TABLE 1:** Procedure‐specific postoperative changes and putative mechanisms contributing to SIBO.

Surgical procedure/setting	Major postoperative anatomical or physiological changes	Potential mechanisms contributing to SIBO	Clinical implications/interpretive notes
Esophagectomy with gastric conduit	Altered foregut anatomy; vagal disruption; impaired motility; altered gastric emptying; reduced oral intake in some patients	Intestinal stasis or dysmotility; altered microbial environment; impaired clearance of bacteria	Breath‐test positivity may be influenced by altered transit; symptoms may overlap with dumping, malabsorption, and anastomotic complications
Gastrectomy (especially Billroth II or Roux‐en‐Y)	Loss of gastric acid barrier; vagotomy; altered reconstruction; blind loop formation; rapid transit	Reduced suppression of ingested bacteria; intestinal stasis in blind loops; altered nutrient flow; dysbiosis	Positive breath tests may be frequent, but accelerated transit may also produce false‐positive findings
Right‐sided colectomy with resection of the ileocecal valve	Loss of ileocecal barrier; altered ileocolonic continuity; possible bile acid malabsorption	Retrograde migration of colonic bacteria into the small bowel	SIBO may coexist with BAM; positive breath tests should be interpreted together with evaluation for BAM
Subtotal colectomy/major colorectal resection	Altered bowel continuity; changes in transit; altered microbiota	Dysbiosis and altered intestinal transit; possible bacterial overgrowth in selected patients	Symptoms such as diarrhea and bloating are nonspecific; other causes should be carefully excluded
Pancreaticoduodenectomy/pancreatic resection	Upper gastrointestinal reconstruction; altered motility; pancreatic exocrine insufficiency; impaired mixing of food with bile and pancreatic secretions	Dysmotility; maldigestion‐related luminal changes; altered intestinal milieu favoring bacterial overgrowth	Direct evidence remains limited; EPI and SIBO may coexist and produce overlapping symptoms
Biliary cancer surgery/biliary reconstruction	Altered bile flow; gastrointestinal reconstruction in selected cases	Altered antimicrobial effects of bile and disturbed intestinal physiology	Evidence remains sparse; biological plausibility exceeds direct clinical evidence

Abbreviations: BAM, bile acid malabsorption; EPI, exocrine pancreatic insufficiency; SIBO, small intestinal bacterial overgrowth.

## 3 Diagnosis of SIBO

Small intestinal bacterial overgrowth (SIBO) is commonly diagnosed using quantitative culture of small intestinal aspirates and breath testing. Quantitative culture of small intestinal fluid, traditionally regarded as the gold standard for SIBO diagnosis, involves obtaining duodenal or jejunal aspirates during upper gastrointestinal endoscopy and identifying bacterial concentrations of ≥ 10^3^ colony‐forming units (CFU)/mL [18]. However, this method is invasive and impractical for repeated assessments. In addition, variability in sampling sites and aspirate volumes may affect diagnostic accuracy and limit standardization. In contrast, the glucose breath test (GBT) is a simple and noninvasive diagnostic tool that is widely used in clinical practice. Following oral administration of 75 g of glucose, hydrogen and methane concentrations in exhaled breath are measured at regular intervals. In patients with SIBO, bacterial fermentation of glucose in the small intestine results in the production of hydrogen and methane, which are subsequently detected in the breath [19]. According to current consensus criteria, a rise in breath hydrogen of ≥ 20 ppm above baseline within 90 min after glucose or lactulose administration is generally considered supportive of SIBO. In contrast, a methane concentration of ≥ 10 ppm at any time during breath testing is currently classified as intestinal methanogen overgrowth (IMO), rather than methane‐predominant SIBO, because methane is produced by methanogenic archaea rather than bacteria [7, 19]. Although breath testing is less accurate than quantitative culture of small intestinal aspirates, it remains the most commonly used diagnostic modality because of its simplicity, safety, and patient acceptability. A recent meta‐analysis reported that the sensitivity and specificity of the glucose breath test ranged from 54% to 63% and 83% to 86%, respectively, whereas those of the lactulose breath test ranged from 42% to 52% and 70% to 86%, respectively [20]. Importantly, interpretation of breath testing may be particularly challenging in patients after gastrointestinal surgery. Surgical reconstruction can substantially alter gastrointestinal transit, and accelerated orocecal transit may allow the administered substrate to reach the colon within the conventional diagnostic timewindow. Consequently, early colonic fermentation may be misinterpreted as evidence of SIBO.

The clinical importance of this limitation was demonstrated by Lin and Massey, who performed glucose breath testing with concurrent scintigraphy in 139 patients with suspected SIBO [21]. Among 46 patients with positive breath‐test results, 22 (48%) were classified as false‐positive because the increase in breath hydrogen or methane occurred after the glucose bolus had reached the cecum, indicating colonic rather than small‐intestinal fermentation. Importantly, colonic fermentation accounted for false‐positive results in 65% of patients with a history of upper gastrointestinal surgery, compared with 13% of patients without previous surgery. The mean orocecal transit time was markedly shorter in patients with false‐positive results than in those with true‐positive or negative tests. These findings demonstrate that accelerated transit after upper gastrointestinal surgery can directly inflate apparent breath‐test positivity rates and should therefore be considered when interpreting postoperative studies. This limitation may be particularly relevant after procedures associated with rapid gastric emptying or altered intestinal continuity. In addition, commonly used breath‐test thresholds have largely been established in populations without major surgically altered gastrointestinal anatomy, and their diagnostic performance has not been sufficiently validated in postoperative cancer patients. Accordingly, breath‐test results after gastrointestinal cancer surgery should be interpreted cautiously in conjunction with the type of surgical reconstruction, gastrointestinal symptoms, and, when available, information regarding gastrointestinal transit. More recently, molecular approaches based on 16S ribosomal RNA (rRNA) gene sequencing have been investigated for the assessment of SIBO. These techniques allow comprehensive characterization of the small intestinal microbiota and may provide insights into microbial alterations associated with SIBO. However, the lack of standardized methodologies and diagnostic thresholds currently limits their routine clinical application, and their use remains largely confined to research settings. Because of its noninvasiveness and feasibility, breath testing is widely used in clinical practice. However, the American College of Gastroenterology (ACG) guideline only conditionally suggests glucose or lactulose hydrogen breath testing for the diagnosis of SIBO in symptomatic patients with previous luminal abdominal surgery, based on a very low level of evidence. Therefore, particularly in patients with surgically altered gastrointestinal anatomy, breath‐test results should be interpreted cautiously and in the context of symptoms, surgical reconstruction, and gastrointestinal transit (Table 2) [7].

**TABLE 2:** Diagnostic methods and criteria for small intestinal bacterial overgrowth and intestinal methanogen overgrowth.

Diagnostic method	Substrate/Sample	Diagnostic criteria	Advantages	Limitations
Small intestinal aspirate culture	Duodenal/jejunal aspirate	≥ 10^3^ CFU/mL	Direct assessment; traditionally considered reference standard	Invasive; sampling variability; risk of contamination; difficult to repeat; limited standardization
Glucose breath test (GBT)	Glucose 75 g	H_2_ rise ≥ 20 ppm above baseline within 90 min supports SIBO; CH_4_ ≥ 10 ppm at any time indicates IMO rather than SIBO	Noninvasive; simple; widely used	Limited sensitivity; diagnostic thresholds are not specifically validated in postoperative patients; accelerated postoperative orocecal transit may allow early colonic fermentation and cause false‐positive results
Lactulose breath test (LBT)	Lactulose	Rise in breath H_2_/CH_4_	Noninvasive; evaluates more distal intestine	Lower specificity; strongly influenced by gastrointestinal transit; rapid transit may cause early colonic fermentation and false‐positive interpretation, particularly after gastrointestinal surgery
16S rRNA sequencing	Intestinal samples	No established diagnostic threshold	Detailed microbiome characterization	No standardized methodology; currently mainly research use

The breath‐test protocols used in the postoperative studies varied substantially. Brennan et al. used hydrogen breath testing in patients after esophagectomy or gastrectomy who were evaluated because SIBO was clinically suspected, whereas Savva et al. performed a standard glucose hydrogen breath test in all included patients; detailed substrate doses and positivity thresholds were not consistently reported in these publications [10, 22]. Paik et al. [23] used a glucose hydrogen–methane breath test and defined positivity as an increase in hydrogen or methane of at least 12 ppm above baseline within 60 min. Pérez Aisa et al. [24] also used a glucose hydrogen–methane breath test and additionally analyzed the breath‐test curve at 45 min to reduce potential false‐positive results attributable to accelerated gastrointestinal transit. Novljan et al. [11] administered 25 g of glucose in 200 mL of water and measured breath hydrogen every 20 min for 120 min; an increase of more than 12 ppm above baseline was considered positive. Kim et al. [25] administered 50 g of glucose and measured both hydrogen and methane at 10‐min intervals for 120 min, using study‐specific criteria based on baseline gas concentrations and an increase of more than 12 ppm within 60 min. Deng et al. [26] used 50–80 g of glucose and measured hydrogen every 20 min for 120 min; an increase of more than 12 ppm was considered positive. Larsen et al. [16] measured both hydrogen and methane and considered an increase of ≥ 10 ppm diagnostic; notably, gastrointestinal transit time was independently assessed using radiopaque markers. Bhagat and Rao [27] used glucose breath testing and/or duodenal aspirate culture in their small symptomatic case series. In the pancreatic resection study by Bromley‐Dulfano et al. [12] glucose breath testing was performed in only eight selected patients, and detailed breath‐test methodology was not reported. Except for the objective assessment of gastrointestinal transit by Larsen et al., the included studies did not systematically incorporate an independent measurement of orocecal transit time. Therefore, differences in substrate dose, gases measured, diagnostic thresholds, and assessment of gastrointestinal transit should be considered when comparing breath‐test positivity rates across studies. Because several earlier studies classified methane‐positive breath tests as SIBO, their original terminology has been retained when describing study‐specific results; however, under current diagnostic terminology, isolated methane positivity meeting the established threshold should be classified as intestinal methanogen overgrowth (IMO).

### 3.1 Esophageal Cancer and SIBO

Several studies have evaluated SIBO and related malabsorption after esophageal and upper gastrointestinal cancer surgery (Table 3). Heneghan et al. [28] conducted a prospective cohort study evaluating malabsorption and malnutrition after curative esophageal and gastric cancer surgery. Of a consecutive series of 66 patients, 45 disease‐free patients were evaluated during long‐term follow‐up, with a median follow‐up of 23 months. Evidence of SIBO was identified in 38% of patients. Malabsorption was present in 73% of the cohort, and 44% had a fecal elastase level below 200 μg/g. Significant postoperative reductions in body weight, body mass index, fat‐soluble vitamin concentrations, and skeletal muscle index were also observed. Although the study included both esophagectomy and gastrectomy patients and was primarily designed to evaluate postoperative malabsorption rather than the prevalence of SIBO specifically, it provides important prospective evidence that SIBO may coexist with other nutritional and malabsorptive disorders in long‐term survivors after upper gastrointestinal cancer surgery. Brennan et al. [22] evaluated breath‐test positivity and associated clinical factors among patients after upper gastrointestinal surgery, including esophagectomy and gastrectomy. SIBO was diagnosed using a hydrogen breath test (HBT). The HBT was performed with the Gastro + Gastrolyzer (Bedfont Scientific Ltd., UK) after a strict 12‐h fast. Among 87 patients evaluated because SIBO was clinically suspected, 46 (52.9%) were HBT‐positive. Comparison between HBT‐positive and HBT‐negative patients revealed significant differences in sex, alcohol consumption, smoking status, and body mass index (BMI). In contrast, surgical procedure, pathological diagnosis, postoperative complications, and perioperative adjuvant treatment were not significantly associated with SIBO. Multivariate analysis demonstrated that female sex and low BMI were independent risk factors for SIBO. However, because this study included both esophagectomy and gastrectomy patients, the incidence and clinical significance of SIBO specifically after esophagectomy remain unclear. Savva et al. evaluated the occurrence of SIBO after oesophagogastric cancer resection in 45 patients who underwent esophagectomy (*n* = 30) or gastrectomy (*n* = 15) in the United Kingdom [10]. A standard glucose hydrogen breath test (GHBT) using the Gastro+ Gastrolyzer breath analyzer was performed in all patients to assess the prevalence of SIBO. Among the 45 patients, 33 (73.3%) were diagnosed with SIBO. Comparison between SIBO‐positive and SIBO‐negative patients revealed a significant difference only in age. In contrast, time since surgery, receipt of chemotherapy, alcohol consumption, smoking status, proton pump inhibitor use, and body mass index (BMI) were comparable between the two groups. These studies reported relatively high breath‐test positivity rates after esophagogastric cancer surgery, ranging from 52.9% to 73.3%. However, these values should not be interpreted as estimates of the true prevalence of SIBO in the overall postoperative population. In particular, Brennan et al. evaluated patients suspected of having SIBO, introducing a substantial risk of selection bias. In addition, both studies included patients undergoing different upper gastrointestinal surgical procedures, and altered postoperative gastrointestinal transit may have affected breath‐test results. Therefore, the true prevalence and clinical significance of SIBO specifically after esophagectomy remain uncertain. Further prospective studies in unselected esophageal cancer survivors using standardized diagnostic methods are warranted.

**TABLE 3:** Clinical evidence of SIBO after gastrointestinal cancer surgery.

Disease	Study	Patients	Surgery/population	Diagnostic method	Breath‐test positivity	Gastrointestinal symptoms	Factors associated with SIBO	Study limitations
Esophageal and gastric cancer	Heneghan et al. (2015) [28]	45	Esophagectomy, *n* = 30; total/subtotal gastrectomy, *n* = 15; disease‐free survivors	H_2_ breath test	37.8% of total cohort (17/45); 47.2% among patients tested (17/36)	Malabsorption‐related symptoms and nutritional abnormalities were common; SIBO coexisted with other malabsorptive disorders	No SIBO‐specific independent predictors were reported	Mixed esophagectomy/gastrectomy population; HBT performed in only 36/45 patients; detailed breath‐test substrate, dose, and diagnostic threshold not reported; no independent assessment of gastrointestinal transit
Esophagogastric cancer	Brennan et al. (2017) [22]	87	Major upper GI cancer surgery including esophagectomy and gastrectomy	H_2_ breath test	52.9% (46/87)	Testing was performed in patients with symptoms or weight loss suggestive of SIBO/malabsorption	Female sex and low BMI were independent risk factors; surgical procedure and perioperative treatment were not significantly associated with SIBO	Selected population with suspected SIBO; not representative of an unselected postoperative cohort; mixed esophagectomy/gastrectomy population; potential influence of altered postoperative transit on breath‐test results
Esophagogastric cancer	Savva et al. (2022) [9]	45	Esophagectomy 30; gastrectomy 15	Glucose H_2_ breath test	73.3% (33/45)	Patients underwent protocol‐based testing irrespective of current GI symptoms	Age differed between SIBO‐positive and SIBO‐negative patients; time since surgery, chemotherapy, alcohol use, smoking, PPI use, and BMI did not differ significantly	Small sample size; mixed surgical procedures; representativeness of the overall postoperative population uncertain; potential transit‐related false positivity
Gastric cancer	Paik et al. (2011) [23]	76	Gastrectomy for early GC	Glucose H_2_/CH_4_ breath test	77.6% (59/76)	Intestinal symptoms were significantly more frequent in breath‐test‐positive patients	Abdominal fullness/borborygmus during glucose loading was associated with positive testing; no significant differences in sex, age, surgical type, postoperative interval, BMI, or dumping syndrome	Single‐center, relatively small cohort; diagnostic threshold differed from current consensus criteria; postoperative transit was not incorporated into breath‐test interpretation
Gastric cancer	Pérez Aisa et al. (2019) [24]	60	Total or subtotal gastrectomy	Glucose H_2_/CH_4_ breath test	61.6%	No clear relationship between breath‐test positivity and nutritional impairment was demonstrated	No significant association with nutritional parameters; BMI was numerically lower in test‐positive patients	Cohort not restricted to gastric cancer; accelerated transit may cause false‐positive results; study used an additional 45‐min analysis to address this issue
Gastric cancer	Novljan et al. (2023) [10]	37	Subtotal 7; total gastrectomy 30	H_2_ breath test	29.7% (11/37)	No clear association between SIBO and gastrointestinal symptoms was demonstrated	No significant clinicopathological differences between SIBO‐positive and negative patients	Small sample size; heterogeneous surgical procedures; diagnostic criteria differed from other studies; potential effect of altered transit on breath‐test results
Gastric cancer	Kim et al. (2017) [25]	14	Gastrectomy	Glucose H_2_/CH_4_ breath test	71.4% (10/14)	Symptom‐specific findings were not separately reported for the gastric cancer subgroup	Breath‐test positivity was higher after gastrectomy than after hysterectomy, cholecystectomy, or in patients with functional GI disorders	Very small gastric cancer subgroup; secondary subgroup comparison; limited precision of positivity estimate; potential postoperative transit‐related misclassification
Colorectal cancer	Deng et al. (2016) [26]	43	Curative CRC surgery	Glucose H_2_ breath test	41.9%	Breath‐test‐positive patients had more severe GI symptoms, particularly diarrhea; symptoms improved after rifaximin	Breath‐test positivity was higher in postoperative CRC patients than in healthy controls (41.9% vs. 6.7%)	Small, non‐randomized study; healthy rather than postoperative controls; treatment analysis limited to test‐positive patients; diagnostic specificity after surgery uncertain
Colorectal cancer	Bhagat & Rao (2017) [27]	4	Subtotal colectomy for colorectal cancer or suspected malignant colon mass	Glucose breath test and/or duodenal aspirate/culture	4/4	Chronic abdominal pain, gas, bloating, and diarrhea after colectomy	No predictors could be assessed because of the small descriptive case series	Highly selected symptomatic referral population; *n* = 4; no unselected denominator; cannot estimate postoperative prevalence; possible overlap with later reports from the same institution
Colon cancer	Larsen et al. (2023) [12]	64	Right‐sided hemicolectomy; 45 with chronic loose stools and 19 controls	Glucose H_2_/CH_4_ breath test	73% vs. 74%	Breath‐test positivity was not associated with chronic loose stools; BAM was substantially more frequent in symptomatic patients (82% vs. 37%)	No association was observed between breath‐test positivity and chronic loose stools or BAM	Case–control design; selected symptomatic and asymptomatic groups rather than an unselected cohort; very high positivity in both groups raises uncertainty regarding the clinical specificity of breath testing after surgery
Pancreatic cancer/pancreatic resection	Bromley‐Dulfano et al. (2022) [11]	545	Pancreatic resection; 83% underwent pancreaticoduodenectomy; 61% had pancreatic adenocarcinoma	Glucose breath test	7/8 tested patients	GI symptoms occurred in 67% of the overall cohort; only selected symptomatic patients underwent gastroenterological evaluation and breath testing	No SIBO‐specific adjusted predictors were reported	Breath testing performed in only 8 highly selected patients; no systematic screening; mixed pancreatic diseases; cannot estimate population‐level SIBO prevalence

### 3.2 Gastric Cancer and SIBO

Four studies have evaluated SIBO after treatment for gastric cancer (Table 3). Paik et al. [23] evaluated the prevalence of small intestinal bacterial overgrowth (SIBO) in 76 patients who underwent gastrectomy for early gastric cancer in Korea. SIBO was diagnosed using a glucose hydrogen–methane breath test. A diagnosis of SIBO was established when the breath hydrogen or methane concentration increased by at least 12 ppm above the baseline level within 60 min after glucose ingestion. Among the 76 patients, 59 (77.6%) were diagnosed with SIBO. Comparison of patient characteristics between the SIBO‐positive and SIBO‐negative groups revealed that the prevalence of intestinal symptoms was significantly higher in patients with SIBO. In contrast, there were no significant differences in sex, age, type of surgery, postoperative interval, body mass index, or the incidence of dumping syndrome between the two groups. Based on these findings, the authors suggested that positive SIBO breath tests were frequently observed after gastrectomy and might represent a potential therapeutic target for postgastrectomy intestinal symptoms. Pérez Aisa et al. [24] prospectively evaluated 60 patients after gastrectomy using a glucose hydrogen–methane breath test. Positive breath‐test findings were observed in 61.6% of patients. Importantly, because accelerated gastrointestinal transit after gastrectomy may result in false‐positive breath‐test findings, the authors performed an additional analysis of the breath‐test curve at 45 min to minimize this potential effect. Breath‐test positivity was not significantly associated with nutritional parameters, although patients with positive tests tended to have a lower BMI. These findings are particularly relevant because they demonstrate both the frequent occurrence of positive breath tests after gastrectomy and the uncertainty regarding their nutritional and clinical significance. Novljan et al. [11] evaluated the prevalence of SIBO in 37 patients who underwent gastrectomy for gastric cancer in Slovenia, including seven patients who underwent subtotal gastrectomy and 30 who underwent total gastrectomy. Before the test, a baseline breath sample was collected using an H2 breath test device (Lactofan 2 Fischer, Leipzig, Germany). An increase in breath hydrogen concentration of more than 12 ppm above the baseline value within 120 min was considered positive for SIBO. Among the 37 patients, 11 (29.7%) were diagnosed with SIBO. No significant differences in clinicopathological characteristics were observed between the SIBO‐positive and SIBO‐negative groups. The breath‐test positivity rate was 29.7%, which was lower than that reported by Paik et al. Differences in diagnostic criteria and breath test protocols may partly explain the variation in the reported prevalence of SIBO among studies. Kim et al. [25] investigated the prevalence of SIBO in patients who had undergone abdominal surgery, including 14 patients with gastrectomy for gastric cancer. SIBO was diagnosed using a glucose hydrogen–methane breath test. The breath‐test positivity rate in the gastrectomy group was 71.4% (10/14), which was significantly higher than that in patients who underwent hysterectomy or cholecystectomy and in those with functional gastrointestinal disorders. The authors suggested that the loss of gastric acid secretion and altered intestinal motility after gastrectomy may contribute to the development of SIBO. Collectively, these studies demonstrate substantial variability in breath‐test positivity after gastrectomy, ranging from 29.7% to 77.6%. However, differences in study design, patient selection, surgical procedures, postoperative intervals, and breath‐test methodologies limit direct comparisons among studies. Therefore, these positivity rates should not be interpreted as a reliable estimate of the prevalence of SIBO in the overall postgastrectomy population. The available studies were generally small, and substantial heterogeneity existed in patient populations, breath‐test protocols, substrates, and diagnostic thresholds. In addition, altered gastric emptying and intestinal transit after gastrectomy may influence breath‐test results and potentially contribute to false‐positive findings. Therefore, although positive breath tests appear to be frequently observed after gastrectomy, the true prevalence and clinical significance of SIBO in this population remain uncertain. Prospective studies in unselected postgastrectomy patients using standardized diagnostic criteria and appropriate consideration of gastrointestinal transit are required.

### 3.3 Colorectal Cancer and SIBO

Three studies have evaluated SIBO after surgical treatment for colorectal cancer (Table 3). Deng et al. [26] investigated the prevalence of SIBO and the therapeutic effect of rifaximin in postoperative colorectal cancer (CRC) patients. The study included 43 patients who had undergone curative surgery for CRC and 30 healthy controls. SIBO was diagnosed using the glucose hydrogen breath test (GHBT), and gastrointestinal symptoms were assessed using the Gastrointestinal Symptom Score (GISS). SIBO‐positive patients received rifaximin at 1200 mg/day for 10 days. The proportion of patients with a positive GHBT was significantly higher in the postoperative CRC group than in healthy controls (41.9% vs. 6.7%, *p* < 0.05). Patients with SIBO had more severe gastrointestinal symptoms than SIBO‐negative patients, particularly diarrhea. After rifaximin treatment, gastrointestinal symptoms significantly improved, especially diarrhea, although normalization of GHBT was achieved in only 33.3% of patients. The authors concluded that postoperative CRC patients are at increased risk of developing SIBO and that SIBO may contribute to postoperative gastrointestinal symptoms. Larsen et al. [16] conducted a case–control study to investigate the roles of SIBO and bile acid malabsorption (BAM) in chronic loose stools following curative right‐sided hemicolectomy for colon cancer. The study included 45 patients with chronic loose stools (Bristol stool types 6–7) and 19 asymptomatic controls, all of whom had undergone surgery at least 1 year before enrollment. All patients underwent glucose hydrogen/methane breath testing for SIBO, selenium‐75 homocholic acid taurine (SeHCAT) scanning for BAM, and gastrointestinal transit time assessment; serum fibroblast growth factor 19 (FGF19) was also measured in a subgroup. Breath‐test positivity was high in both groups, being detected in 73% of patients with chronic loose stools and 74% of asymptomatic controls, with no significant difference between groups. In contrast, BAM was significantly more common in patients with chronic loose stools than in controls (82% vs. 37%, *p* < 0.001). No association was observed between SIBO and BAM. Although bowel symptoms improved after targeted treatment, the authors concluded that chronic loose stools after right‐sided hemicolectomy were primarily associated with BAM rather than SIBO. Bhagat and Rao reported a case series of four patients who developed chronic abdominal pain, bloating, gas, and diarrhea after subtotal colectomy for colorectal cancer [27]. SIBO was identified using glucose breath testing and/or duodenal aspirate culture, and antibiotic treatment was associated with resolution of symptoms. However, these patients represented a highly selected symptomatic population referred for evaluation of otherwise unexplained gastrointestinal symptoms. Therefore, the 100% positivity observed in this case series cannot be interpreted as an estimate of the prevalence of SIBO after colorectal cancer surgery. A subsequent study from the same institution evaluated a larger cohort of symptomatic patients after colectomy for mixed indications. Because this cohort was not restricted to colorectal cancer and potential overlap with the earlier case series could not be excluded, it was not treated as an independent cancer‐specific cohort in the present review [29].

Taken together, these studies indicate that positive SIBO breath tests may be frequently observed after colorectal cancer surgery, but their clinical significance remains uncertain. Deng et al. reported a higher breath‐test positivity rate in postoperative colorectal cancer patients than in healthy controls and found more severe gastrointestinal symptoms, particularly diarrhea, among test‐positive patients. In contrast, Larsen et al. found nearly identical breath‐test positivity rates in patients with chronic loose stools and asymptomatic postoperative controls (73% vs. 74%), whereas bile acid malabsorption was substantially more frequent in symptomatic patients. These findings suggest that breath‐test positivity alone may not reliably identify the cause of postoperative gastrointestinal symptoms. Other conditions, including bile acid malabsorption and altered gastrointestinal transit, should therefore be considered when interpreting positive breath‐test results after colorectal cancer surgery. Further prospective studies in unselected postoperative populations are needed to determine the true prevalence and clinical relevance of SIBO in this setting.

### 3.4 Pancreatobiliary Cancer Surgery and SIBO

Pancreatobiliary surgery may also theoretically predispose patients to SIBO. Pancreaticoduodenectomy results in substantial alterations in gastrointestinal anatomy and physiology, including reconstruction of the upper gastrointestinal tract, altered intestinal transit and motility, exocrine pancreatic insufficiency (EPI), and impaired coordination between food passage and biliary and pancreatic secretions. These changes provide a plausible pathophysiological basis for bacterial overgrowth.

However, direct clinical evidence remains limited. Bromley‐Dulfano et al. retrospectively evaluated 545 patients after pancreatic resection, of whom 61% underwent surgery for pancreatic adenocarcinoma. Gastrointestinal symptoms were reported in 67% of patients, but only 105 symptomatic patients were referred for gastroenterological evaluation [12]. Glucose breath testing was performed in only eight patients, seven of whom were diagnosed with SIBO. Because breath testing was restricted to a highly selected subgroup of symptomatic patients, these findings cannot be used to estimate the prevalence of SIBO in the overall population after pancreatic resection.

Evidence regarding SIBO after biliary cancer surgery is even more limited. Although clinical guidelines for cholangiocarcinoma recognize SIBO as a potential cause of gastrointestinal symptoms after surgical resection, robust prospective studies using systematic SIBO testing are lacking. Therefore, despite a plausible biological rationale, current evidence is insufficient to establish patients undergoing pancreatic or biliary cancer surgery as a high‐risk postoperative population for SIBO. Prospective studies using predefined diagnostic criteria and systematic postoperative testing are required.

### 3.5 Postoperative Differential Diagnoses and Nutritional Impact

Postoperative gastrointestinal symptoms such as diarrhea, abdominal bloating, steatorrhea, and weight loss are nonspecific and should not automatically be attributed to SIBO. Important differential diagnoses include bile acid malabsorption (BAM), EPI, dumping syndrome, lactose or other carbohydrate intolerance, medication‐related diarrhea, gastrointestinal infection, postoperative stricture or mechanical obstruction, and cancer recurrence. Moreover, SIBO, BAM, and EPI are not mutually exclusive and may coexist or produce overlapping symptoms. In a systematic review of malabsorption following oesophagectomy, Khaw et al. [30] identified EPI, SIBO, and BAM as important postoperative malabsorption syndromes, although definitions and diagnostic methods varied considerably among studies. Diagnostic evaluation should therefore be guided by the predominant clinical features and surgical anatomy. BAM can be assessed using the selenium‐75 homocholic acid taurine (^75^SeHCAT) retention test or, where available, fasting serum 7α‐hydroxy‐4‐cholesten‐3‐one (C4). However, ^75^SeHCAT has limited availability and involves radiation exposure, whereas serum C4 is influenced by diurnal variation and requires a fasting morning sample; liver disease may also affect its interpretation [31, 32]. EPI is commonly assessed using fecal elastase‐1 because of its simplicity and noninvasive nature. However, its sensitivity is limited in mild EPI, and watery diarrhea may dilute fecal elastase concentrations and produce falsely low values; therefore, low values obtained from watery stools should be interpreted cautiously and repeated when appropriate [33]. Structural causes such as anastomotic stricture, bowel obstruction, or cancer recurrence require targeted radiological or endoscopic evaluation according to the clinical context.

From a biological perspective, SIBO may contribute to nutritional impairment through bacterial competition for vitamin B12 and bacterial deconjugation of bile acids, which can impair micelle formation and fat absorption. Severe SIBO may therefore be associated with vitamin B12 deficiency, steatorrhea, and deficiencies of fat‐soluble vitamins [7, 18]. These mechanisms could theoretically aggravate postoperative weight loss and loss of skeletal muscle mass. However, postoperative malnutrition and sarcopenia are multifactorial, and direct evidence establishing SIBO as an independent cause of these outcomes after gastrointestinal cancer surgery is lacking. Notably, Pérez Aisa et al. found no significant association between breath‐test positivity and nutritional parameters after gastrectomy, although a lower BMI was observed numerically in test‐positive patients [24]. Therefore, the potential contribution of SIBO to postoperative weight loss, sarcopenia, impaired tolerance of cancer treatment, delayed recovery, or long‐term survival should currently be regarded as biologically plausible rather than clinically established.

### 3.6 Cancer‐Specific Effect Modifiers and Potential Confounders

Several cancer‐ and treatment‐related factors may influence both the development and ascertainment of SIBO after gastrointestinal cancer surgery. Chemotherapy and radiotherapy can cause gastrointestinal symptoms and alter mucosal integrity and the intestinal microbiota, potentially confounding associations between breath‐test positivity and postoperative symptoms. PPIs reduce gastric acid secretion and have been associated with an increased risk of SIBO, whereas antibiotics can substantially alter the intestinal microbiota and breath hydrogen and methane production. Opioids may impair gastrointestinal motility and promote intestinal stasis. In addition, differences in oral intake and enteral or parenteral nutritional support may modify the intestinal environment, although direct evidence regarding their effects on postoperative SIBO remains limited. These exposures should therefore be regarded as potential effect modifiers or confounders rather than established causes of SIBO in postoperative cancer patients. Reporting of these factors was incomplete in the available studies. Savva et al. evaluated chemotherapy and PPI use and found no significant differences between patients with positive and negative glucose hydrogen breath tests; patients who had received antibiotics within the preceding 4 weeks were excluded [10]. However, the duration of PPI exposure, a specific PPI washout period, and multivariable adjustment for PPI use were not reported. In most other postoperative studies, information regarding radiotherapy, opioid exposure, nutritional support, duration of PPI treatment, and medication washout before breath testing was limited or absent. Consequently, residual confounding by these factors cannot be excluded and should be considered when interpreting the reported associations between SIBO testing, symptoms, and postoperative outcomes.

### 3.7 Proposed Clinical Approach to Suspected SIBO After Gastrointestinal Cancer Surgery

To improve the clinical applicability of this review, a pragmatic approach to suspected postoperative SIBO may be considered. Patients with persistent diarrhea, bloating, excessive gas, steatorrhea, weight loss, or nutritional abnormalities should first be evaluated in the context of the surgical procedure and reconstruction. Important alternative or coexisting disorders, including bile acid malabsorption, exocrine pancreatic insufficiency, dumping syndrome, infection, medication‐related diarrhea, postoperative obstruction or stricture, and cancer recurrence, should be considered before attributing symptoms to SIBO. Breath testing may be considered when SIBO remains clinically suspected, although the results should be interpreted cautiously because altered postoperative anatomy and accelerated gastrointestinal transit may reduce diagnostic specificity. Treatment should be directed toward the most likely underlying mechanism, followed by reassessment of symptoms and nutritional status. Persistent or recurrent symptoms should prompt reconsideration of alternative or coexisting diagnoses. Because high‐quality interventional evidence in postoperative cancer patients remains limited, this proposed approach should be regarded as a pragmatic, expert‐opinion‐based framework rather than a validated management algorithm.

## 4 Treatment of SIBO

Evidence regarding the treatment of SIBO specifically after gastrointestinal cancer surgery remains limited. For clinical interpretation, the available therapeutic evidence can be categorized according to its directness into three groups: (1) studies directly involving postoperative patients, (2) studies involving patients with cancer but not restricted to postoperative populations, and (3) indirect evidence derived from general SIBO populations. Direct postoperative evidence is limited, and we identified no primary therapeutic study specifically representing the second category. Consequently, most available evidence regarding antibiotics, probiotics, elemental diets, and recurrence prevention is derived from general SIBO populations without major surgically altered gastrointestinal anatomy and should be considered indirect when applied to patients after gastrointestinal cancer surgery. In the general SIBO population, antibiotic therapy is the most extensively studied treatment approach, with rifaximin being the most frequently evaluated agent. Direct evidence in patients after gastrointestinal cancer surgery is considerably more limited. Direct postoperative evidence is available from only a small number of studies. Deng et al. evaluated rifaximin in postoperative colorectal cancer patients with positive glucose hydrogen breath tests. After rifaximin at 1200 mg/day for 10 days, overall gastrointestinal symptom scores improved significantly, particularly for diarrhea. In contrast, breath‐test normalization was achieved in only 6 of 18 patients (33.3%) at the end of the 10‐day treatment period [26]. Thus, symptomatic improvement and breath‐test normalization were not equivalent treatment outcomes in this study. In another postoperative study, Pérez Aisa et al. prospectively evaluated antibiotic treatment in postgastrectomy patients with positive glucose hydrogen–methane breath tests. Breath‐test positivity persisted in 94.6% of patients after rifaximin and in 85.7% after subsequent metronidazole, and the overall antibiotic treatment failure rate was 67.6% [24]. Nutritional parameters were reassessed 1 month after successful treatment, but no significant improvement was observed. These findings demonstrate that eradication of breath‐test positivity may be particularly difficult after gastrectomy and highlight the limitations of extrapolating treatment efficacy reported in general SIBO populations to patients with surgically altered gastrointestinal anatomy. We did not identify primary treatment studies specifically involving patients with gastrointestinal cancer without restriction to the postoperative setting; therefore, evidence of intermediate directness between postoperative studies and general SIBO populations is currently lacking. Although these findings suggest that rifaximin may provide symptomatic benefit in selected postoperative patients, the small sample size, non‐randomized design, and reliance on breath‐test positivity limit definitive conclusions regarding its efficacy in this population. The remaining therapeutic evidence is indirect and is derived predominantly from general SIBO populations. A systematic review and meta‐analysis by Shah et al. [34] identified ten prospective clinical trials evaluating antibiotic therapy for SIBO (Table 4). Overall, antibiotics were superior to placebo for breath test normalization, with pooled normalization rates of 51.1% for antibiotics compared with 9.8% for placebo. Rifaximin was the most frequently studied antibiotic and achieved an overall breath test normalization rate of approximately 50%, although treatment efficacy varied considerably according to study population, dosage, and treatment regimen. Furthermore, normalization of the breath test was generally associated with greater symptomatic improvement, suggesting that bacterial eradication correlates with clinical response. However, the available evidence is limited by small sample sizes and heterogeneous study designs, and the optimal antibiotic regimen has not yet been established. Therefore, further high‐quality randomized controlled trials are required to determine the most effective treatment strategy for SIBO.

**TABLE 4:** Current evidence for the treatment of small intestinal bacterial overgrowth.

Treatment category	Study	Study population	Surgical status	Directness of evidence	Treatment/intervention	Main outcome	Key findings
Antibiotics	Shah et al. (2013) [34]	Meta‐analysis; 10 prospective trials	Not surgery‐specific	Indirect	Various antibiotics	Breath test normalization	**51.1%** with antibiotics vs. **9.8%** with placebo
Rifaximin	Gatta et al. (2017) [35]	Systematic review/meta‐analysis; 32 studies, 1331 patients	Predominantly non‐postoperative/not surgery‐specific	Indirect	Rifaximin	SIBO eradication	**70.8%** eradication; adverse events **4.6%**
Various single‐ and multi‐strain probiotic formulations; strains, doses, and treatment durations varied across studies	Zhong et al. (2017) [36]	Systematic review/meta‐analysis; 18 studies	Not surgery‐specific	Indirect	Probiotics	SIBO decontamination	Improved decontamination (**RR 1.61**, 95% CI 1.19–2.17); improved abdominal pain
Elemental diet	Pimentel et al. (2004) [37]	93 patients	Non‐postoperative/not surgery‐specific	Indirect	Elemental diet for 14–21 days	Breath test normalization	**80% at 14 days**, increasing to **85% at 21 days**
Rifaximin after CRC surgery	Deng et al. (2016) [26]	Postoperative CRC patients with SIBO	Postoperative cancer patients	Direct postoperative evidence	Rifaximin 1200 mg/day × 10 days	GHBT normalization/symptoms	GHBT normalization **33.3%**; GI symptoms, particularly diarrhea, improved
Antibiotics after gastrectomy	Pérez Aisa et al. (2019) [24]	Patients after gastrectomy with positive glucose H_2_–CH_4_ breath tests	Postoperative patients	Direct postoperative evidence	Rifaximin followed by metronidazole in persistent cases	Persistent breath‐test positivity/treatment failure	High persistence of breath‐test positivity; overall antibiotic treatment failure 67.6%
Recurrence after rifaximin	Lauritano et al. (2008) [38]	Prospective; 80 successfully treated patients	Not surgery‐specific	Indirect	Rifaximin 1200 mg/day × 7 days	SIBO recurrence	**12.6% at 3 months, 27.5% at 6 months, 43.7% at 9 months**

*Note:* Direct postoperative evidence refers to studies specifically evaluating treatment in patients after gastrointestinal cancer surgery. No primary therapeutic study involving patients with gastrointestinal cancer without restriction to the postoperative setting was identified. Therefore, most evidence regarding antibiotics, probiotics, elemental diets, and recurrence is indirect and derived from general, non‐surgery‐specific SIBO populations. The bold values indicate statistically significant results (*p* < 0.05).

Among the available antibiotics, rifaximin, a minimally absorbed oral antibiotic with broad‐spectrum activity against aerobic and anaerobic bacteria, has been the most extensively investigated. In a systematic review and meta‐analysis including 32 studies and 1331 patients, Gatta et al. [35] reported an overall SIBO eradication rate of 70.8% (95% CI, 61.4%–78.2%) in the intention‐to‐treat analysis, while symptomatic improvement was observed in approximately two‐thirds of patients with successful eradication. In addition, rifaximin was well tolerated, with an overall adverse event rate of only 4.6%. However, these data were derived predominantly from non‐postoperative SIBO populations, and their applicability to patients after gastrointestinal cancer surgery remains uncertain.

In addition to antibiotics, probiotics have been explored as an alternative or adjunctive treatment for SIBO. A systematic review and meta‐analysis by Zhong et al. [30] included 18 studies and demonstrated that probiotics significantly increased the SIBO decontamination rate compared with control treatment (RR 1.61, 95% CI 1.19–2.17). Probiotic therapy also significantly reduced hydrogen concentrations on breath testing and improved abdominal pain, although no significant benefit was observed in preventing SIBO or reducing stool frequency. Notably, the combination of probiotics and antibiotics achieved a higher decontamination rate than probiotics alone (85.8% vs. 53.2%), suggesting that probiotics may be more effective as an adjunct rather than as a replacement for antibiotic therapy. The probiotic interventions included in this meta‐analysis were heterogeneous and comprised both single‐strain and multi‐strain preparations. Reported formulations included *Lactobacillus fermentum* KLD, Lactobacillus species, combinations of bifidobacteria and lactobacilli, and multi‐strain preparations containing *Lactobacillus casei* , *Lactobacillus plantarum* , *Streptococcus faecalis* , and Bifidobacterium brevis; treatment regimens ranged from several days to several months, and probiotic doses were inconsistently reported across studies [30]. However, the considerable heterogeneity among studies regarding probiotic strains, dosage, treatment duration, and patient populations limits definitive conclusions. Importantly, because probiotic effects are strain‐ and formulation‐specific, efficacy observed with a particular probiotic preparation cannot necessarily be generalized to other strains, combinations, doses, or treatment regimens. In addition, evidence specifically evaluating probiotics in patients with SIBO after gastrointestinal cancer surgery is lacking.

Nutritional therapy, particularly elemental diet, has also been proposed as a treatment strategy for SIBO. Pimentel et al. [37] evaluated a 14‐day exclusive elemental diet in 93 patients with abnormal lactulose breath tests suggestive of SIBO. Breath test normalization was achieved in 80% of patients after 14 days and increased to 85% after extending treatment to 21 days in selected patients. Patients who achieved breath test normalization also experienced significantly greater improvement in gastrointestinal symptoms than those with persistent SIBO. These findings suggest that elemental diet may be effective in selected patients with abnormal breath tests. However, this evidence was not derived from postoperative gastrointestinal cancer patients, and its applicability to patients with surgically altered gastrointestinal anatomy remains uncertain.

Despite these treatment options, recurrence remains common in SIBO after treatment. Recurrence after successful antibiotic therapy remains a major challenge in the long‐term management of SIBO. Lauritano et al. [38] prospectively evaluated 80 patients with SIBO who achieved normalization of the glucose breath test (GBT) after rifaximin treatment (1200 mg/day for 7 days). During follow‐up, SIBO recurrence was observed in 12.6%, 27.5%, and 43.7% of patients at 3, 6, and 9 months, respectively. Multivariate analysis identified older age, a history of appendectomy, and chronic proton pump inhibitor (PPI) use as independent risk factors for recurrence. Moreover, recurrence of GBT positivity was accompanied by relapse of gastrointestinal symptoms, suggesting that recurrent bacterial overgrowth is clinically relevant. These findings indicate that recurrence after successful antibiotic therapy is common in general SIBO populations. However, the recurrence rate and associated risk factors after gastrointestinal cancer surgery remain largely unknown.

## 5 Limitations of the Current Evidence

The available evidence regarding SIBO after gastrointestinal cancer surgery has several important methodological limitations. First, the included studies are heterogeneous in design, including prospective and retrospective cohorts, case–control studies, and small case series. Several studies evaluated selected symptomatic patients or individuals referred for breath testing because SIBO or malabsorption was clinically suspected, rather than consecutive unselected postoperative populations. Consequently, the reported breath‐test positivity rates may be substantially affected by selection and referral bias and should not be interpreted as estimates of the true postoperative prevalence of SIBO. Second, sample sizes were generally small, and in some reports only a small subset of the source population underwent diagnostic testing. Third, adjustment for potential confounding factors was inconsistent, and information regarding missing data and time from surgery was incompletely reported in some studies. Finally, substantial heterogeneity existed in breath‐test substrates, diagnostic thresholds, sampling intervals, and positivity criteria. These methodological differences, together with surgery‐related alterations in gastrointestinal transit, limit comparisons across studies and the generalizability of the currently available evidence. Accordingly, well‐designed prospective studies enrolling consecutive postoperative patients and applying standardized diagnostic protocols are required to determine the true prevalence and clinical significance of SIBO after gastrointestinal cancer surgery.

## 6 Future Perspectives

Although increasing evidence suggests that small intestinal bacterial overgrowth (SIBO) may occur after gastrointestinal cancer surgery, several important issues remain unresolved. First, standardization of SIBO diagnosis is essential [7, 20, 21]. Breath testing is widely used in clinical studies because it is non‐invasive and feasible; however, diagnostic protocols, substrates, sampling intervals, and positivity criteria vary substantially among studies. This heterogeneity makes it difficult to compare reported breath‐test positivity rates across esophageal, gastric, and colorectal cancer surgery.

Second, optimization of breath‐test criteria is particularly important in postoperative patients [4, 7, 20, 21]. Surgical reconstruction may substantially alter gastrointestinal transit, and accelerated orocecal transit can allow the test substrate to reach the colon within the conventional diagnostic time window, potentially resulting in false‐positive findings. Blind loops, reduced gastric acid secretion, and loss of the ileocecal valve may further influence breath‐test results. Therefore, criteria established in non‐surgical populations may not be directly applicable to patients after gastrointestinal cancer surgery. Future studies should validate postoperative‐specific glucose or lactulose breath‐test criteria and, where possible, incorporate objective assessment of gastrointestinal transit. Third, microbiome‐based approaches may provide deeper insight into the pathophysiology of postoperative SIBO [4, 6, 17]. Comprehensive analyses of the gut microbiota, bile acid metabolism, short‐chain fatty acids, and inflammatory profiles may help identify patients at high risk of SIBO and clarify the relationship between dysbiosis, abdominal symptoms, nutritional impairment, and postoperative recovery. Fourth, optimization of rifaximin therapy is needed [7, 34, 35, 38]. Although rifaximin is one of the most extensively studied antibiotics for SIBO, the optimal dose, duration, retreatment strategy, and patient selection remain unclear in gastrointestinal cancer surgery. Prospective studies are required to determine whether rifaximin improves not only breath test normalization but also abdominal symptoms, nutritional status, body composition, quality of life, and long‐term postoperative outcomes. Fifth, recurrence prevention and maintenance therapy represent important future challenges [7, 34, 35, 36]. SIBO frequently recurs after antibiotic treatment, particularly when underlying anatomical or functional abnormalities persist. Strategies such as probiotics, prebiotics, dietary intervention, prokinetic therapy, or microbiome‐targeted maintenance therapy may be useful, but evidence in postoperative cancer patients remains limited. Well‐designed prospective studies are warranted. Finally, SIBO should be considered as one of several potential postoperative disorders within the broader field of gastrointestinal cancer surgery [4, 7, 18]. As surgical procedures become increasingly individualized and survivorship care becomes more important, appropriate recognition and evaluation of suspected SIBO may contribute to improved management of postoperative gastrointestinal symptoms, nutritional impairment, and quality of life. Future multidisciplinary research integrating surgery, gastroenterology, nutrition, microbiome science, and oncology will be essential to establish evidence‐based diagnostic and therapeutic strategies for SIBO after gastrointestinal cancer surgery.

## 7 Conclusion

SIBO should be considered as an important differential diagnosis in symptomatic patients after gastrointestinal cancer surgery. Although available studies have reported relatively frequent breath‐test positivity after esophageal, gastric, and colorectal cancer surgery, these findings should not be interpreted as reliable estimates of the true postoperative prevalence of SIBO because of patient selection, small study populations, heterogeneous diagnostic criteria, and altered gastrointestinal transit. Evidence after pancreatobiliary cancer surgery is even more limited. In addition, direct evidence regarding treatment in postoperative cancer patients remains scarce, and most therapeutic data are extrapolated from general SIBO populations. Therefore, the true prevalence, clinical significance, and optimal diagnostic and management strategies for SIBO after gastrointestinal cancer surgery remain to be established.

## Author Contributions

**Toru Aoyama:** conceptualization, methodology, investigation, writing – original draft, writing – review and editing. **Haruhiko Cho:** investigation, writing – review and editing. Takaki Yoshikawa: conceptualization, supervision, writing – review and editing. All authors have read and approved the final manuscript.

## Funding

The authors have nothing to report.

## Ethics Statement

The authors have nothing to report.

## Consent

The authors have nothing to report.

## Conflicts of Interest

The authors declare no conflicts of interest.

## Supporting information


**Table S1:** PubMed/MEDLINE search strategy.
**Table S2:** Methodological characteristics of studies evaluating SIBO after gastrointestinal cancer surgery.

## References

[1] F. Bray , M. Laversanne , H. Sung , et al., “Global Cancer Statistics 2022: GLOBOCAN Estimates of Incidence and Mortality Worldwide for 36 Cancers in 185 Countries,” CA: A Cancer Journal for Clinicians 74 (2024): 229–263. 10.3322/caac.21834 38572751

[2] E. Scarpellini , J. Arts , G. Karamanolis , et al., “International Consensus on the Diagnosis and Management of Dumping Syndrome,” Nature Reviews. Endocrinology 16 (2020): 448–466. 10.1038/s41574-020-0357-5 PMC7351708 32457534

[3] J. L. Davis and R. T. Ripley , “Postgastrectomy Syndromes and Nutritional Considerations Following Gastric Surgery,” Surgical Clinics of North America 97 (2017): 277–293. 10.1016/j.suc.2016.11.005 28325187

[4] D. Bushyhead and E. M. M. Quigley , “Small Intestinal Bacterial Overgrowth‐Pathophysiology and Its Implications for Definition and Management,” Gastroenterology 163 (2022): 593–607. 10.1053/j.gastro.2022.04.002 35398346

[5] J. Bures , J. Cyrany , D. Kohoutova , et al., “Small Intestinal Bacterial Overgrowth Syndrome,” World Journal of Gastroenterology 16 (2010): 2978–2990. 10.3748/wjg.v16.i24.2978 PMC2890937 20572300

[6] U. C. Ghoshal , R. Shukla , U. Ghoshal , K. A. Gwee , S. C. Ng , and E. M. M. Quigley , “The Gut Microbiota and Irritable Bowel Syndrome: Friend or Foe?,” International Journal of Inflammation 2012 (2012): 151085. 10.1155/2012/151085 PMC3346986 22577594

[7] M. Pimentel , R. J. Saad , M. D. Long , and S. S. C. Rao , “ACG Clinical Guideline: Small Intestinal Bacterial Overgrowth,” American Journal of Gastroenterology 115 (2020): 165–178. 10.14309/ajg.0000000000000501 32023228

[8] A. Mechlińska , K. Frąckiewicz , K. Gładyś‐Cieszyńska , D. Buczek , and R. Dziadziuszko , “Small Intestinal Bacterial Overgrowth and Intestinal Methanogen Overgrowth in Gastrointestinal Malignancies,” Contemporary Oncology (Pozn) 29 (2025): 11–21, 10.5114/wo.2025.148643. PMC12051882 40330452

[9] K. V. Savva , L. Hage , I. Belluomo , P. Gummet , P. R. Boshier , and C. J. Peters , “Assessment of the Burden of Small Intestinal Bacterial Overgrowth (SIBO) in Patients After Oesophagogastric (OG) Cancer Resection,” Journal of Gastrointestinal Surgery 26 (2022): 924–926. 10.1007/s11605-021-05177-w PMC9021082 34671913

[10] U. Novljan and T. Pintar , “Small Intestinal Bacterial Overgrowth After Total and Subtotal Gastrectomy; High Incidence—Not Connected to Gastrointestinal Symptoms,” Gastroenterologist 2 (2023): 12–26.

[11] R. Bromley‐Dulfano , A. T. August , A. Y. Li , W. Park , and B. Visser , “Characterizing Gastrointestinal Dysfunction After Pancreatic Resection: A Single‐Center Retrospective Study,” BMC Gastroenterology 22 (2022): 488, 10.1186/s12876-022-02565-7. PMC9701409 36435757

[12] H. M. Larsen , K. Krogh , M. Borre , et al., “Chronic Loose Stools Following Right‐Sided Hemicolectomy for Colon Cancer and the Association With Bile Acid Malabsorption and Small Intestinal Bacterial Overgrowth,” Colorectal Disease 25 (2023): 600–607. 10.1111/codi.16409 36347822

[13] A. C. Dukowicz , B. E. Lacy , and G. M. Levine , “Small Intestinal Bacterial Overgrowth: A Comprehensive Review,” Gastroenterol Hepatol (N Y) 3 (2007): 112–122. PMC3099351 21960820

[14] E. Husebye , “Gastrointestinal Motility Disorders and Bacterial Overgrowth,” Journal of Internal Medicine 237 (1995): 419–427, 10.1111/j.1365-2796.1995.tb01196.x. 7714466

[15] E. Grace , C. Shaw , K. Whelan , and H. J. Andreyev , “Review Article: Small Intestinal Bacterial Overgrowth—Prevalence, Clinical Features, Current and Developing Diagnostic Tests, and Treatment,” Alimentary Pharmacology & Therapeutics 38 (2013): 674–688. 10.1111/apt.12456 23957651

[16] W. K. Lo and W. W. Chan , “Proton Pump Inhibitor Use and the Risk of Small Intestinal Bacterial Overgrowth: A Meta‐Analysis,” Clinical Gastroenterology and Hepatology 11 (2013): 483–490, 10.1016/j.cgh.2012.12.011. 23270866

[17] V. Maksimaityte , A. Bausys , M. Kryzauskas , et al., “Gastrectomy Impact on the Gut Microbiome in Patients With Gastric Cancer: A Comprehensive Review,” World Journal of Gastrointestinal Surgery 13 (2021): 678–688. 10.4240/wjgs.v13.i7.678 PMC8316847 34354801

[18] E. M. M. Quigley , J. A. Murray , and M. Pimentel , “AGA Clinical Practice Update on Small Intestinal Bacterial Overgrowth: Expert Review,” Gastroenterology 159 (2020): 1526–1532. 10.1053/j.gastro.2020.06.090 32679220

[19] A. Rezaie , M. Buresi , A. Lembo , et al., “Hydrogen and Methane‐Based Breath Testing in Gastrointestinal Disorders: The North American Consensus,” American Journal of Gastroenterology 112 (2017): 775–784. 10.1038/ajg.2017.46 PMC5418558 28323273

[20] G. Losurdo , G. Leandro , E. Ierardi , et al., “Breath Tests for the Non‐Invasive Diagnosis of Small Intestinal Bacterial Overgrowth: A Systematic Review With Meta‐Analysis,” Journal of Neurogastroenterology and Motility 26 (2020): 16–28. 10.5056/jnm19113 PMC6955189 31743632

[21] E. C. Lin and B. T. Massey , “Scintigraphy Demonstrates High Rate of False‐Positive Results From Glucose Breath Tests for Small Bowel Bacterial Overgrowth,” Clinical Gastroenterology and Hepatology 14 (2016): 203–208. 10.1016/j.cgh.2015.07.032 26241509

[22] M. Brennan , M. Fanning , A. Granahan , et al., “Small Intestinal Bacterial Overgrowth in Patients Post Major Upper Gastrointestinal Cancer Surgery,” Diseases of the Esophagus 30 (2017): 1–7.

[23] C. N. Paik , M. G. Choi , C. H. Lim , et al., “The Role of Small Intestinal Bacterial Overgrowth in Postgastrectomy Patients,” Neurogastroenterology and Motility 23 (2011): e191–e196. 10.1111/j.1365-2982.2011.01686.x 21324050

[24] A. Pérez Aisa , M. C. García Gavilán , J. Alcaide García , et al., “Small Intestinal Bacterial Overgrowth Is Common After Gastrectomy but With Little Impact on Nutritional Status,” Gastroenterología y Hepatología 42 (2019): 1–10, 10.1016/j.gastrohep.2018.07.001. 30197248

[25] D. B. Kim , C. N. Paik , Y. J. Kim , et al., “Positive Glucose Breath Tests in Patients With Hysterectomy, Gastrectomy, and Cholecystectomy,” Gut Liver 11 (2017): 237–242. 10.5009/gnl16132 PMC5347648 27965476

[26] L. Deng , Y. Liu , D. Zhang , Y. Li , and L. Xu , “Prevalence and Treatment of Small Intestinal Bacterial Overgrowth in Postoperative Patients With Colorectal Cancer,” Molecular and Clinical Oncology 4 (2016): 883–887. 10.3892/mco.2016.807 PMC4840787 27123301

[27] J. W. Bhagat and S. S. C. Rao , “Small Intestinal Bacterial Overgrowth Following Subtotal Colectomy for Colon Cancer,” Colorec Cancer 3 (2017): 13, 10.21767/2471-9943.100043.

[28] H. M. Heneghan , A. Zaborowski , M. Fanning , et al., “Prospective Study of Malabsorption and Malnutrition After Esophageal and Gastric Cancer Surgery,” Annals of Surgery 262 (2015): 807–808, 10.1097/SLA.0000000000001445. 26583669

[29] S. S. C. Rao , G. Tan , H. Abdulla , S. Yu , S. Larion , and P. Leelasinjaroen , “Does Colectomy Predispose to Small Intestinal Bacterial (SIBO) and Fungal Overgrowth (SIFO)?,” Clinical and Translational Gastroenterology 9, no. 4 (2018): 146, 10.1038/s41424-018-0011-x. PMC5915536 29691369

[30] R. A. Khaw , E. J. Nevins , and A. W. Phillips , “Incidence, Diagnosis and Management of Malabsorption Following Oesophagectomy: A Systematic Review,” Journal of Gastrointestinal Surgery 26 (2022): 1781–1790, 10.1007/s11605-022-05323-y. 35484473

[31] R. P. Arasaradnam , S. Brown , A. Forbes , et al., “Guidelines for the Investigation of Chronic Diarrhoea in Adults: British Society of Gastroenterology, 3rd Edition,” Gut 67 (2018): 1380–1399, 10.1136/gutjnl-2017-315909. PMC6204957 29653941

[32] P. Vijayvargiya , M. Camilleri , A. Shin , and A. Saenger , “Methods for Diagnosing Bile Acid Malabsorption: A Systematic Review,” BMC Gastroenterology 19 (2019): 185, 10.1186/s12876-019-1102-1. PMC6854889 31726982

[33] M. E. Phillips , A. D. Hopper , J. S. Leeds , et al., “Consensus for the Management of Pancreatic Exocrine Insufficiency: UK Practical Guidelines,” BMJ Open Gastroenterology 8 (2021): e000643, 10.1136/bmjgast-2021-000643. PMC8212181 34140324

[34] S. C. Shah , L. W. Day , M. Somsouk , and J. L. Sewell , “Meta‐Analysis: Antibiotic Therapy for Small Intestinal Bacterial Overgrowth,” Alimentary Pharmacology & Therapeutics 38 (2013): 925–934. 10.1111/apt.12479 PMC3819138 24004101

[35] L. Gatta and C. Scarpignato , “Systematic Review With Meta‐Analysis: Rifaximin Is Effective and Safe for the Treatment of Small Intestine Bacterial Overgrowth,” Alimentary Pharmacology & Therapeutics 45 (2017): 604–616. 10.1111/apt.13928 PMC5299503 28078798

[36] C. Zhong , C. Qu , B. Wang , S. Liang , and B. Zeng , “Probiotics for Preventing and Treating Small Intestinal Bacterial Overgrowth: A Meta‐Analysis and Systematic Review of Current Evidence,” Journal of Clinical Gastroenterology 51, no. 4 (2017): 300–311, 10.1097/MCG.0000000000000814. 28267052

[37] M. Pimentel , T. Constantino , Y. Kong , M. Bajwa , A. Rezaei , and S. Park , “A 14‐Day Elemental Diet Is Highly Effective in Normalizing the Lactulose Breath Test,” Digestive Diseases and Sciences 49 (2004): 73–77. 10.1023/b:ddas.0000011605.43979.e1 14992438

[38] E. C. Lauritano , M. Gabrielli , E. Scarpellini , et al., “Small Intestinal Bacterial Overgrowth Recurrence After Antibiotic Therapy,” American Journal of Gastroenterology 103 (2008): 2031–2035, 10.1111/j.1572-0241.2008.02030.x. 18802998

