# Supplementary material for: Small Intestinal Bacterial Overgrowth Following Gastrointestinal Cancer Surgery: Current Evidence and Future Perspectives
Source: Ann Gastroenterol Surg. 2026 Oct 1:10.1002/ags3.70291. Online ahead of print. doi: 10.1002/ags3.70291 (PMC13629109; doi:10.1002/ags3.70291)
Supplement: Supplementary file 1 [file AGS3-9999-0-s001.docx]

**Supplementary Table S1. PubMed/MEDLINE search strategy**

| **Search** | **Search terms** |
| --- | --- |
| #1 SIBO | "small intestinal bacterial overgrowth"[Title/Abstract] OR "small bowel bacterial overgrowth"[Title/Abstract] OR SIBO[Title/Abstract] |
| #2 Esophageal/gastric surgery | esophagectomy[Title/Abstract] OR oesophagectomy[Title/Abstract] OR gastrectomy[Title/Abstract] OR "esophageal cancer"[Title/Abstract] OR "oesophageal cancer"[Title/Abstract] OR "gastric cancer"[Title/Abstract] OR "gastrointestinal surgery"[Title/Abstract] OR "gastrointestinal cancer"[Title/Abstract] OR "gastrointestinal malignancy"[Title/Abstract] |
| #3 Colorectal surgery | "colorectal surgery"[Title/Abstract] OR colectomy[Title/Abstract] OR hemicolectomy[Title/Abstract] OR "colorectal cancer"[Title/Abstract] OR "colon cancer"[Title/Abstract] OR "rectal cancer"[Title/Abstract] |
| #4 Pancreatobiliary surgery | pancreatectomy[Title/Abstract] OR pancreaticoduodenectomy[Title/Abstract] OR pancreatoduodenectomy[Title/Abstract] OR "pancreatic cancer"[Title/Abstract] OR "biliary surgery"[Title/Abstract] OR "biliary cancer"[Title/Abstract] OR cholangiocarcinoma[Title/Abstract] |
| **#5 Final search** | **#1 AND (#2 OR #3 OR #4)** |

**Database:** PubMed/MEDLINE
**Search period:** From database inception through July 1, 2026
**Date of final search:** July 1, 2026
**Language restriction:** None
**Other filters:** None
**Additional searching:** Reference lists of eligible articles and relevant reviews were manually screened, and citation tracking was performed to identify additional potentially eligible studies, including directly relevant reports not indexed in PubMed.

**Supplementary Table S2. Methodological characteristics of studies evaluating SIBO after gastrointestinal cancer surgery**

| **Study** | **Study design** | **Sampling frame / denominator** | **Indication for testing** | **Time from surgery** | **Breath-test protocol** | **PPI exposure / medication washout** | **Adjustment / relevant confounders** |
| --- | --- | --- | --- | --- | --- | --- | --- |
| **Heneghan et al. (2015)** | Prospective cohort study | Consecutive series of 66 patients undergoing curative esophageal or gastric cancer surgery; 45 disease-free patients constituted the study cohort; 30 esophagectomy and 15 total/subtotal gastrectomy | Protocol-based assessment of malabsorption and nutritional status in disease-free survivors; not restricted to patients referred because of suspected SIBO | Minimum follow-up ≥18 months; median follow-up 23.1 months (range 18–28); HBT performed at 6 months postoperatively | Hydrogen breath testing performed in 36/45 patients (80%) at 6 months; 17 patients were positive. Substrate, dose, sampling interval, and positivity threshold were not specified in the report | PPI exposure and specific medication washout before HBT not reported | Malabsorption, EPI, BAM, nutritional parameters, body composition, and multimodal cancer treatment were assessed. Multivariable analysis identified advanced tumor stage as associated with overall malabsorption, but no SIBO-specific adjusted predictor analysis was reported |
| **Brennan et al. (2017)** | Retrospective review | 87 patients undergoing HBT after esophagectomy or gastrectomy; selected patients with suspected postoperative malabsorption/SIBO | Weight loss or symptoms suggestive of malabsorption/SIBO | Patients tested <6 months, 6–12 months, or >1 year after surgery | Glucose hydrogen breath test; detailed dose and positivity threshold NR in the published report | PPI exposure/washout NR | Multivariable regression performed; female sex and lower BMI independently associated with positive HBT; surgical procedure and perioperative treatment were also examined |
| **Savva et al. (2022)** | Prospective cross-sectional observational study | 190 patients approached; 45 eligible and tested (30 esophagectomy, 15 gastrectomy), irrespective of current GI symptoms | Protocol-based testing, not symptom-triggered | ≥1 year after surgery; 7.59±2.71 years in GHBT− vs 7.89±3.50 years in GHBT+ patients | Standard glucose hydrogen breath test; substrate dose and detailed positivity threshold NR | PPI use reported in both groups (5 GHBT− and 17 GHBT+ patients); duration/washout NR. Antibiotic use within previous 4 weeks excluded | Univariate comparisons of age, BMI, chemotherapy, PPI use, smoking, alcohol use, and interval from surgery; no multivariable adjustment reported |
| **Paik et al. (2011)** | Prospective study | 76 patients after gastrectomy for early gastric cancer without recurrence; protocol-based testing | Systematic study assessment rather than testing for suspected SIBO | Postoperative interval assessed; exact summary interval NR in accessible report | Oral glucose H₂–CH₄ breath test; rise in H₂ or CH₄ ≥12 ppm above baseline within 60 min | PPI exposure/washout NR | Clinical characteristics and dumping-related variables assessed; abdominal fullness/borborygmus during glucose loading independently predicted positive testing |
| **Pérez Aisa et al. (2019)** | Prospective cohort study | 60 patients after total or subtotal gastrectomy; cohort not restricted to gastric cancer | Protocol-based postoperative evaluation | ≥3 months after gastrectomy | Glucose H₂–CH₄ breath test; additional analysis of the curve at 45 min to reduce potential false positivity from rapid transit | PPI exposure/washout NR | Demographic and nutritional parameters assessed; SIBO positivity was not significantly associated with nutritional parameters |
| **Novljan et al. (2023)** | Observational analytical cross-sectional study | 37 patients after curative total (n=30) or subtotal (n=7) gastrectomy for gastric cancer; patients with or without GI symptoms | Systematic study testing | Mean approximately 25 months after surgery; broad postoperative range | 25 g glucose in 200 mL water; H₂ measured every 20 min for 120 min; rise >12 ppm above baseline considered positive | PPI exposure NR; antibiotics, prokinetics, and laxatives withheld according to study protocol | Symptoms, anthropometric variables, dietary patterns, and neoadjuvant/adjuvant chemotherapy or radiotherapy evaluated; no significant associations with SIBO reported |
| **Kim et al. (2017)** | Retrospective chart review / cross-sectional analysis | 171 patients with previous abdominal surgery; gastric cancer subgroup comprised 14 gastrectomy patients | Patients undergoing GBT as part of clinical evaluation; not an unselected gastrectomy cohort | Gastric cancer patients had ≥6 months of recurrence-free follow-up | 50 g glucose; H₂ and CH₄ measured at baseline and every 10 min for 120 min; study-specific criteria included baseline elevation or >12-ppm increase within 60 min | PPIs, H₂-receptor antagonists, antibiotics, probiotics, laxatives, prokinetics, narcotics, and antidiarrheal drugs within previous 4 weeks excluded | Comparisons among surgical and control groups; no SIBO-specific multivariable adjustment reported |
| **Deng et al. (2016)** | Observational postoperative cohort with treatment of test-positive patients | 43 postoperative CRC patients and 30 healthy controls; all CRC patients underwent GHBT | Protocol-based postoperative testing | NR | 50–80 g glucose in 200–250 mL water; H₂ measured every 20 min for 120 min; >12-ppm increase considered positive | PPI exposure/washout NR | SIBO-positive and SIBO-negative patients compared for GI symptoms; no multivariable adjustment reported |
| **Bhagat & Rao (2017)** | Case series | 4 highly selected patients referred to a motility center after subtotal colectomy for colorectal cancer or suspected malignant colonic mass | Chronic unexplained abdominal pain, gas, bloating, and/or diarrhea after otherwise negative evaluation | Variable; chronic postoperative symptoms, with individual cases occurring long after surgery | 75 g glucose in 250 mL water; samples every 15 min for 2 h; >20 ppm H₂ or >10 ppm CH₄ considered positive; and/or distal duodenal aspirate culture ≥10³ CFU/mL | PPI exposure NR; no narcotics, probiotics, or antibiotics during previous 6 weeks | Descriptive case series; no adjustment for confounders; highly selected referral population |
| **Larsen et al. (2023)** | Case–control study | 45 patients with chronic loose stools and 19 postoperative controls without loose stools after curative right-sided hemicolectomy | Cases selected for chronic loose stools; controls selected without loose stools; all subsequently underwent systematic testing | Median 3.8 years (range 1.0–13.0) in cases and 5.9 years (1.5–15.2) in controls | Glucose H₂/CH₄ breath test; ≥10-ppm increase considered positive; GITT independently measured using radiopaque markers | PPI-specific exposure/washout NR; daily medications reviewed | Adjuvant chemotherapy similar between groups (43% vs 42%); BAM, SIBO and GITT systematically assessed; no association between breath-test positivity and chronic loose stools |
| **Bromley-Dulfano et al. (2022)** | Retrospective single-center cohort | 545 patients after pancreatic resection; 366/545 reported postoperative GI symptoms; 105 referred to gastroenterology; only 8 underwent glucose breath testing | Breath testing performed in a highly selected subgroup referred for postoperative GI dysfunction | GI symptoms commonly developed early after surgery; exact interval from surgery to GBT NR | Glucose breath test; substrate dose, gases measured, and positivity criteria NR | 132 symptomatic patients (36%) received PPI therapy; timing relative to GBT, duration, and washout NR | Demographic and operative factors evaluated for GI dysfunction/referral; no SIBO-specific adjusted analysis; only 8 patients underwent breath testing |

**Abbreviations:** BAM, bile acid malabsorption; BMI, body mass index; CFU, colony-forming units; CH₄, methane; CRC, colorectal cancer; GBT, glucose breath test; GHBT, glucose hydrogen breath test; GI, gastrointestinal; GITT, gastrointestinal transit time; H₂, hydrogen; HBT, hydrogen breath test; NR, not reported; PPI, proton pump inhibitor; SIBO, small intestinal bacterial overgrowth.
**NR indicates that the relevant information was not reported in the original publication. Study-specific historical terminology for methane-positive breath tests is retained; under current terminology, isolated methane positivity meeting established criteria would be classified as intestinal methanogen overgrowth (IMO).**
